# Status of adult inpatient burn rehabilitation in Europe: are we neglecting metabolic outcomes?

**DOI:** 10.1093/burnst/tkaa039

**Published:** 2021-03-01

**Authors:** David R Schieffelers, Eric van Breda, Nick Gebruers, Jill Meirte, Ulrike Van Daele

**Affiliations:** 1 Multidisciplinary Metabolic Research Unit (M2RUN), MOVANT Research Group, Department of Rehabilitation Sciences and Physiotherapy, Faculty of Medicine and Health Sciences, University of Antwerp, Universiteitsplein 1, 2610, Wilrijk, Antwerp, Belgium; 2 Multidisciplinary Edema Clinic, Antwerp University Hospital, Wilrijkstraat 10, 2650, Edegem, Antwerp, Belgium; 3 OSCARE, Organisation for burns, scar after-care and research, Van Roiestraat 18, 2170 Merksem, Antwerp, Belgium

**Keywords:** Burn care, Burn rehabilitation, Exercise, Early mobilization, Hypermetabolism, Adults

## Abstract

**Background:**

Hypermetabolism, muscle wasting and insulin resistance are challenging yet important rehabilitation targets in the management of burns. In the absence of concrete practice guidelines, however, it remains unclear how these metabolic targets are currently managed. This study aimed to describe the current practice of inpatient rehabilitation across Europe.

**Methods:**

An electronic survey was distributed by the European Burn Association to burn centres throughout Europe, comprising generic and profession-specific questions directed at therapists, medical doctors and dieticians. Questions concerned exercise prescription, metabolic management and treatment priorities, motivation and knowledge of burn-induced metabolic sequelae. Odds ratios were computed to analyse associations between data derived from the responses of treatment priorities and knowledge of burn-induced metabolic sequelae.

**Results:**

Fifty-nine clinicians with 12.3 ± 9 years of professional experience in burns, representing 18 out of 91 burn centres (response rate, 19.8%) across eight European countries responded. Resistance and aerobic exercises were only provided by 42% and 38% of therapists to intubated patients, 87% and 65% once out-of-bed mobility was possible and 97% and 83% once patients were able to leave their hospital room, respectively. The assessment of resting energy expenditure by indirect calorimetry, muscle wasting and insulin resistance was carried out by only 40.7%, 15.3% and 7.4% respondents, respectively, with large variability in employed frequency and methods. Not all clinicians changed their care in cases of hypermetabolism (59.3%), muscle wasting (70.4%) or insulin resistance (44.4%), and large variations in management strategies were reported. Significant interdisciplinary variation was present in treatment goal importance ratings, motivation and knowledge of burn-induced metabolic sequelae. The prevention of metabolic sequelae was regarded as the least important treatment goal, while the restoration of functional status was rated as the most important. Knowledge of burn-induced metabolic sequelae was linked to higher importance ratings of metabolic sequelae as a therapy goal (odds ratio, 4.63; 95% CI, 1.50–14.25; *p* < 0.01).

**Conclusion:**

This survey reveals considerable non-uniformity around multiple aspects of inpatient rehabilitation across European burn care, including, most notably, a potential neglect of metabolic outcomes. The results contribute to the necessary groundwork to formulate practice guidelines for inpatient burn rehabilitation.

Highlights

- Burn-induced metabolic derangements are challenging yet important rehabilitation targets in the successful management of burns. Early goal-directed rehabilitation has the potential to ameliorate metabolic derangements.
- European burn clinicians were surveyed to identify the current practice of inpatient rehabilitation across Europe.
- Resistance and aerobic exercises are not consistently provided in the early phase.
- Metabolic outcomes are under-used as therapeutic and assessment targets.
- Restoring functional status, not metabolic sequelae, is regarded as the most important therapeutic goal.
- Few burn clinicians demonstrated knowledge of post-burn metabolic pathophysiology.

## Background

Continuing advances in post-burn care have progressively shifted the focus from mere survival towards long-term improvements in overall health and quality of life [1]. Among other significant challenges to these long-term outcomes are long-lasting derangements in glucose, lipid and protein metabolism. These burn-induced metabolic derangements are key drivers of the development of post-burn hypermetabolism, a state of increased metabolic rate and one of the hallmarks of the stress response after burns [2]. The stress response entails two distinct phases of metabolic regulation. The first 24–48 hours of burn injury are known as the “ebb” phase, during which cardiac output, oxygen consumption, metabolism and glucose tolerance are markedly reduced [3]. This is followed by the “flow” phase, which is characterized by gradual increases in cardiac output, oxygen consumption, metabolism and catabolism [4, 5]. Together with prolonged periods of immobilization, these metabolic derangements contribute to persistent muscle wasting and insulin resistance, both of which hamper full recovery and may place the burn survivor at a higher risk of developing cardiovascular and metabolic comorbidities long after the initial burn trauma [2, 4–11]. Long-term comorbidities in turn pose substantial challenges to burn survivorship, impeding full return to work and reintegration into society [12, 13].

Significant research efforts over the past three decades have shed more light into the pathophysiological processes underlying the post-burn stress response and its detrimental effects on energy expenditure, skeletal muscle catabolism and glycaemic control [14]. This progressively increasing pathophysiological understanding has given rise to the development of interventions aimed at ameliorating associated metabolic outcomes. However, many questions as to the optimal management of hypermetabolism, muscle wasting and insulin resistance remain unanswered.

Among interventions that have been proposed to alter the course of burn-induced metabolic sequelae is exercise-based rehabilitation [15–19]. Accumulating evidence for the restorative effects of exercise, in particular in the paediatric burn population, has led international practice guidelines to recommend exercise regimens to be routinely incorporated into the long-term rehabilitation of burn survivors post-hospital discharge [20–22]. Favourable results of exercise, when commenced after hospital discharge, include an increase in lean mass, muscle strength, aerobic capacity and quality of life [20, 23, 24].

During the acute in-hospital phase, however, guidance concerning exercise is still in its infancy and there is little evidence regarding its effects [20–25]. The latest practice guidelines published by the International Society of Burn Injuries include the early institution of exercise as a part of the recommended metabolic management for the first time, but without concrete advice for exercise components [22]. It is during the acute phase that burn-induced metabolic sequelae are most prevalent and that exercise training might be most potent. In particular, aerobic and resistance exercise, as highly potent forms of metabolic stimuli [26, 27], could be key components in the early management of metabolic sequelae after burns. However, it remains unclear to what degree different types of exercise, as well as medical or nutritional interventions, are currently used in clinical practice for the purpose of optimizing metabolic outcomes.

Following overwhelming evidence in other critical illnesses [28], where early rehabilitative approaches have been long implemented (for their ability to resist metabolic sequelae [29–33], amongst other reasons), burn clinicians are increasingly adopting early rehabilitative approaches into their standard care [34–36]. Despite this clinical trend, prescribed exercise parameters, such as exercise type, intensity, timing and the physiological foundations upon which they are built, remain ill-defined in the absence of concrete exercise guidelines for adult burns. Recent findings from a large-scale study of exercise practice in both adult and paediatric burn patients confirm non-uniformity in the use of exercise and choice of exercise type in the acute phase of burns, both in the intensive care unit (ICU) and post-ICU prior to complete wound healing [36]. A key factor that might explain the non-uniformity in choice and use of early exercise is the clinician’s perceived relative importance of different therapeutic goals. Perceived goal importance, in turn, is largely informed by the clinician’s knowledge of burn pathophysiology and the perceived rationale of various types of exercise. However, to date, these factors have not yet been explored in burn clinicians in relation to clinical decision making in exercise rehabilitation.

Defining the role of metabolic outcomes in adult inpatient burn rehabilitation will serve to inform steps forward in developing practice guidelines aimed at creating more conformity. This study was therefore initiated to survey the European burn care community in order to provide insight into the current status of (1) inpatient exercise rehabilitation; (2) the management of hypermetabolism, muscle wasting and insulin resistance; and (3) treatment priorities, motivation and knowledge.

## Methods

Upon approval by the Institutional Review Board of the Ziekenhuis Netwerk Antwerpen/OCMW, an electronic survey was distributed by the European Burn Association to burn centres across Europe, directed at burn clinicians, including physiotherapists and occupational therapists (from here on referred to as therapists), medical doctors and dieticians. Questions were designed and recorded using a transport layer-secure encrypted online platform (Qualtrics; LCC, USA).

The questions aimed to identify the following three components of current practice concerning the rehabilitation of adult patients with burns encompassing ≥20% total body surface area (TBSA): (1) inpatient exercise prescription, including exercise provision and components, inclusion/exclusion criteria, exercise parameters and the influence of metabolic sequelae; (2) metabolic management, including the evaluation and treatment of hypermetabolism, muscle wasting and insulin resistance; and (3) treatment priorities, motivation and knowledge, including therapeutic goal importance, exercise rationale and the clinician’s knowledge of burn-induced metabolic sequelae. The cut-off point of ≥20% TBSA was chosen as metabolic sequelae have been well documented in this adult patient population [37–40].

The survey (S1) comprised both generic and profession-specific questions in the form of multiple-choice and open questions. Generic questions concerned clinician and burn centre characteristics, as well as the clinician’s treatment priorities, motivation and knowledge (i.e. the third survey component as described above). Profession-specific questions for therapists primarily related to exercise prescription (first survey component), whereas medical doctors and dieticians answered questions largely concerned with the metabolic management (second survey component). Exercise provision and components across different phases of inpatient stay were determined through a “constant sum question” type (see Q21-23 in S1). According to this question type, therapists were asked to allocate a percentage of the total treatment time they spent per patient to prespecified treatment components, with the sum totaling 100%. The prevalence of provision of treatment components was determined through binary coding of the obtained responses into equal to (i.e. non-prevalent) or higher than (i.e. prevalent) 0% of allocated total treatment duration. Survey questions that investigated the management of hypermetabolism, muscle wasting and insulin resistance asked participants to report which, if any, outcome measures and intervention strategies they used, and at which frequency, for each respective outcome. Prespecified answer options and an open text field were used for questions regarding the type of outcome measures, whereas questions concerning the type of intervention strategies contained open text fields. To assess treatment priorities, all participants were asked to rate 5 pre-specified treatment goals over the acute phase of burns on a Likert scale of importance. The rated goals were:

(1) range of motion (join mobility, skin mobility);
(2) scar quality (aesthetics, pruritus, pain, prevention of hypertrophic scarring);
(3) restoration of functional status (activities of daily living, ambulation ability, etc.);
(4) prevention of deconditioning (muscle weakness, cardiovascular deconditioning, etc.); and
(5) prevention of metabolic sequelae (insulin resistance, hyperglycaemia, fat and muscle catabolism, etc.).

Therapists were additionally asked to list (in descending priority) the reasons why they thought active exercise should be included in the acute phase of burns. Responses were grouped according to common therapeutic goals. To avoid suggestive cues, this question was asked prior to the aforementioned treatment goal importance ratings. Knowledge of burn-related metabolic pathophysiology was assessed by asking all survey participants to list short- and long-term metabolic effects occurring after burns. Entered responses were grouped according to common keywords (e.g. hypermetabolism or elevated metabolic rate or other deviations of the same term) and scored as present or absent knowledge in the following categories: ebb phase, flow phase, hypermetabolism, hyperglycaemia, insulin resistance and hypercatabolism. To ensure the validity of the responses, participants were asked not to consult additional resources as these questions assessed ad hoc (i.e. readily available) knowledge, as it is this knowledge that is mostly applicable to daily clinical practice).

The survey flow made use of a display logic method to skip or display questions based on those previously answered. Using this display logic, the number of questions posed to therapists ranged between 18 and 29, or 17 and 22 for medical doctors and dieticians. All but a small number of open questions required a response to progress. Survey structure and content were informed by a review of current evidence, including a comparable survey [41] and author expertise (UVD, DRS). Overall, it was estimated that survey completion would take participants 15–30 minutes.

**Table 1:** Percentage of total treatment duration per treatment component per phase of inpatient stay

	**ROM**	**Resistance**	**Aerobic**	**Proprioception**	**Function**	**Respiratory**
While intubated (%)^a^	95.8 (23)	41.7 (10)	37.5 (9)	25.0 (6)	50.0 (12)	83.3 (20)
OOB mobility allowed (%)^b^	96.8 (30)	87.1 (27)	64.5 (20)	41.9 (13)	90.3 (28)	45.2 (17)
Allowed to leave room (%)^c^	100.0 (30)	96.7 (29)	83.3 (25)	76.7 (23)	93.3 (28)	20.0 (6)

^a^total number of responders, n=24; ^b^total number of responders, n=31; ^c^total number of responders, n=30. *OOB* out of bed mobility, *ROM* Range of motion exercises

To ensure that questions were correctly understood, the survey was first conducted in Belgian and Dutch burn centres with one of the authors (DRS) present during data collection. The survey was then distributed by the European Burn Association to all burn centres within the European Burn Association’s email contact database (91 burn centres in 28 European countries). Burn centres had to be listed in the European Burn Association’s email contact database to be eligible for survey distribution. This database comprises centres providing any form of services for inpatient burn care. Participants were eligible if they worked in burn centres at the time of survey participation; were therapists, medical doctors, dieticians or nurses involved in inpatient burn care; and treated adult burns. Participants that exclusively treated paediatric burns or only worked with outpatients were excluded from participation.

Email instructions were used to direct the survey to the respective burn clinicians within the institutions. As survey invitations were sent by the European Burn Association on the behalf of the authors of this study, it was impossible to verify how many email contacts were active, or to carry out a non-responder analysis.

Following its distribution in June 2018 and a reminder email 40 days later, the online survey remained active for 8 months. Partial responders received an automatic email reminder 1 week after an incomplete survey had been recorded, and unless completed within 30 days were otherwise excluded from analysis.

Complete responses were coded and exported to Microsoft Excel (Microsoft, USA) and, where appropriate, measures of distribution were calculated and presented as means ± 95% CIs. Associations were analysed between the following variables: respondent’s profession, importance ratings and knowledge of the flow phase of metabolic sequelae. Recoding of non-binary variables into binary data was carried out where low counts in some categories did not allow meaningful analysis. Accordingly, the importance ratings on the Likert scale were recoded into extremely important versus all other importance ratings. Odds ratios (ORs) were computed and associations were tested with the Fisher’s exact test using SPSS Statistics Version 25 (IBM, USA). The significance level was set at *p* = 0.05.

## Results

### Characteristics

Overall, 64 burn clinicians responded to the survey, out of which 5 participants were excluded from analysis due to incomplete responses. All of the 5 excluded participants (3 medical doctors and 2 occupational therapists from the UK and The Netherlands) did not progress beyond 15% of the survey, which mostly equates with completely the demographics section. The remaining 59 clinicians (32 therapists (30 physiotherapists, 2 occupational therapists), 19 medical doctors and 8 dieticians), representing 18 out of 91 burn centres (19.8% response rate) across eight European countries (Belgium, Portugal, Slovakia, Spain, Sweden, Switzerland, the Netherlands and the United Kingdom) completed the survey and gave informed consent. The average years of professional experience in burns amongst participants was 12.3 ± 9 SD years.

### Exercise prescription

#### Exercise provision and components

All therapists stated that they used some form of active exercise (defined as any independent or assisted muscular activity involving skeletal muscle contractions) in their respective burn centre; however, only less than half of these categorically reported commencing resistance (41.7%) or aerobic exercise (37.5%) in intubated patients. The provision of resistance and aerobic exercise increased to 87.1% and 64.5% once out-of-bed mobility was possible, and to 96.7% and 83.3% once patients were able to leave their hospital room, respectively (Table 1).

The relative proportion of total treatment time that was allocated to resistance and aerobic exercise increased over the course of the hospital stay, however, this varied considerably between therapists (Table 2). The largest proportions of total treatment time across the different phases were for treatment aimed at preserving/restoring joint range of motion, with functional training making up the second-largest proportion once out-of-bed mobility was established.

**Table 2:** 

	**ROM**	**Resistance**	**Aerobic**	**Proprioception**	**Function**	**Respiratory**
While intubated (%)	48.8 (36.5-61)	6.5 (2.6-10.3)	4.8 (1.7-7.9)	2.7 (0-5.5)	8.8 (3.9-13.6)	26.9 (18.9-34.9)
OOB mobility allowed (%)	33.1 (25.9-40.2)	16.5 (12.8-20.1)	11.5 (7.7-15.2)	4.4 (2.1-6.6)	26 (18.4-33.6)	7.4 (3.8-11)
Allowed to leave room (%)	28.8 (23.2-34.4)	21.0 (17.4-24.6)	16.8 (13.2-20.4)	8.2 (5.7-10.6)	22.6 (17.7-27.5)	2.7 (0.1-5.2)

Data presented as mean (95%CI). *OOB* out of bed mobility, *ROM* Range of motion exercises

**Figure 1.**
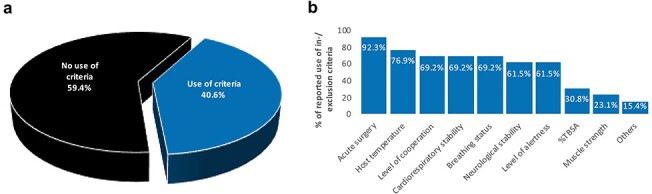
Predefined in^−^/exclusion criteria for active exercise. (**a**) Reported use; (**b**) Frequency of reported in-/exclusion criteria given by those that reported use. *TBSA* total body surface area

#### Inclusion and exclusion criteria

Predefined inclusion/exclusion criteria for active exercise varied greatly among therapists and were only used by 40.6% of respondents (Figure 1). Of those 40.6%, the most common inclusion/exclusion criteria for active exercise were acute surgery (92.3%), host temperature (76.9%), cardiorespiratory stability (69.2%), breathing status (69.2%), level of cooperation (69.2%), neurological status (61.5%) and level of alertness (6.5%). Less frequently used criteria were %TBSA (30.8%), muscle strength (23.1%) and others (15.4%). Of those that reported not using any criteria, 35.7% stated that they carried out active exercise only if prescribed by a doctor.

#### Exercise parameters

The method used to determine exercise intensity varied considerably amongst respondents, with patient tolerance (68.8%), heart rate (12.5%), general exercise guidelines (12.5%) (without specifying employed methods) and V̇O_2_ max (3.1%) being reported for aerobic exercise. Other methods (12.5%) used included the therapist's intuition, trial and error, haemodynamics and respiratory rate.

For resistive exercise, intensity was primarily based on patient tolerance (59.4%) and manual muscle testing (28.1%). The repetition maximum (12.5%), dynamometry (9.4%) and other methods (21.9%) were used less frequently by therapists, with other methods including the therapist’s intuition and the functional status of the patient. Muscle groups targeted as part of the resistance exercise also varied, with the whole body, upper limbs, lower limbs, the core or the burned location being trained by 43.8%, 65.6%, 68.8%, 50% and 15.6% of therapists, respectively.

The majority of therapists (96.7%) stated that they did not work with an overall fixed-length exercise programme. Patient-dependent factors, such as goal achievement (31.3%), hospital discharge (37.5%) and burn unit discharge (6.3%), determined when exercise programmes were discontinued, or instead continued after hospital discharge in an outpatient setting (15.6%). The advice to follow an exercise programme after hospital discharge was given by most therapists either categorically (62.5%) or depending on the patient (34.4%).

#### Influence of metabolic sequelae

The post-burn development of hyperglycaemia, insulin resistance or a hypermetabolic state did not change the majority (87.5%) of therapists’ exercise prescription. The main reasons given by therapists for this were: (1) a lack of understanding of the metabolic sequelae (50%); (2) it is not their responsibility (21.9%); and (3) a lack of understanding the effects of exercise on these parameters (15.6%).

### Metabolic management

The use of outcome measures to assess energy expenditure, muscle wasting, insulin sensitivity and muscle force is summarized in Table 3. Table 4 gives an overview of the reported intervention strategies for the burn-induced development of hypermetabolism, muscle wasting and insulin resistance. For additional data presented per profession the reader is referred to Table S2 and Table S3 in the supplementary material.

**Table 3:** Outcome measures

**Outcome**	**Methods**	**% (frequency)**	**Applied frequency**	**% (frequency)**
**Energy expenditure**	Indirect calorimetry (IC)	40.7 (11)	Daily	3.7 (1)
*Respondents:*	Via mechanical ventilator	40.7 (11)	Weekly	25.9 (7)
Medical doctors and dieticians (n = 27)	Spontaneous breathing	3.7 (1)	Biweekly	0 (0)
	Criteria indicating use of IC		Only when indicated	11.1 (3)
	Mechanical ventilation	40.7 (11)		
	%TBSA	14.8 (4)		
	Unexplained weight loss	11.1 (3)		
	Other metabolic issue	3.7 (1)		
	Prediction formulas	88.9 (24)	Daily	25.9 (7)
	Toronto	37 (10)	Weekly	33.3 (9)
	Fixed kcal/kg	18.5 (5)	Biweekly	7.4 (2)
	Harris–Benedict	14.8 (4)	Only when indicated	22.2 (6)
	Curreri	14.8 (4)		
	Others^a^	14.8 (4)		
**Muscle wasting**	Not measured	84.7 (50)	Daily	0 (0)
*Respondents:*	Body-weight monitoring	11.9 (7)	Weekly	11.9 (7)
Medical doctors, dieticians and therapists (n = 59)	Eye judgement of muscle volume	5.1 (3)	Biweekly	0 (0)
	Muscle force assessment	3.4 (2)	Only when indicated	3.4 (2)
	Muscle circumference	1.7 (1)		
	Nitrogen Balance	1.7 (1)		
	Bioimpedance Analysis	1.7 (1)		
**Insulin sensitivity**	Not measured	92.6 (25)	Daily	0 (0)
*Respondents:*	HOMA-IR	3.7 (1)	Weekly	3.7 (1)
Medical doctors and dieticians (n = 27)	ISI	3.7 (1)	Biweekly	0 (0)
			Only when indicated	3.7 (1)
**Muscle force**	Not measured	40.6 (13)	Daily	3.1 (1)
*Respondents:*	Manual muscle testing	46.9 (15)	Weekly	28.1 (9)
Therapists (n = 32)	Handheld dynamometry	31.3 (10)	Biweekly	9.4 (3)
	Indirectly through functional tests	25 (8)	Only when indicated	18.8 (6)
	Isokinetic dynamometry	3.1 (1)		

^a^Including Henry’s, Milner, Garland, Xi. *TBSA* total body surface area, *HOMA-IR* homeostasis model assessment of insulin resistance, *ISI* Insulin Sensitivity Index

**Table 4:** Metabolic interventions

**Therapeutic target**	**Intervention**	**% (frequency)**
**Hypermetabolism** ^a^	No strategy	40.7 (11)
*Respondents:*	Modify nutrition^b^	59.3 (16)
Medical doctors and dieticians (n = 27)	Betablockers	44.4 (12)
	Early coverage/grafting	40.7 (11)
	Anabolic steroids	37 (10)
	Glycaemic control	29.6 (8)
	Early excision	25.9 (7)
	Adapt ambient temperature	22.2 (6)
	Exercise	11.1 (3)
	Infection control	11.1 (3)
	Others^c^	18.5 (5)
**Muscle wasting**	No strategy	29.6 (8)
*Respondents:*	Exercise	66.7 (18)
Medical doctors and dieticians (n = 27)	Modify nutrition	55.6 (15)
	Anabolic steroids	14.8 (4)
	Betablockers	7.4 (2)
	Limit duration/depth of sedation	7.4 (2)
	Others^d^	11.1 (3)
**Insulin sensitivity**	No strategy	55.6 (15)
*Respondents:*	Insulin infusion	48.1 (13)
Medical doctors and dieticians (n = 27)	Moderate glycaemic control	25.9 (7)
	Tight glycaemic control	22.2 (6)
	Hypoglycaemic diet	7.4 (2)
	Avoid overfeeding	7.4 (2)
	Anabolic steroids	7.4 (2)
	Early excision	7.4 (2)
	Exercise	7.4 (2)
	Others^e^	18.5 (5)

^a^Defined as >10% predicted resting energy expenditure; ^b^including increasing and decreasing caloric provision, supplementing nutrition content (protein, trace elements, vitamins) early enteral feeding; ^c^including fenofibrates, growth hormones, early resuscitation, limiting sedation, anxiety reduction; ^d^including fenofibrates, avoiding neuromuscular blockers, early excision, early coverage; ^e^including Gliclazide, Metformin, betablockers, fenofibrates, early coverage

#### Energy expenditure

The use of predictive formulas to estimate energy expenditure in burn patients was more common (88.9%) than the use of indirect calorimetry (40.7%). Of those that used indirect calorimetry, all did so via a mechanical ventilator, with only one respondent reporting methods during spontaneous breathing independent of a mechanical ventilator. Energy expenditure determination was mostly reported to be carried out on a weekly basis or only when indicated. The most common indication criteria for indirect calorimetry were mechanical ventilation, %TBSA and unexplained weight loss. The classical Toronto formula was most often used (37%) to estimate energy requirements. Less frequently mentioned formulas were fixed kcal/kg (18.5%), Harris–Benedict (14.8%) and Curreri (14.8%).

Employed strategies to manage the hypermetabolic response after burns varied widely, with 40.7% reporting no strategy whatsoever. The most common strategies were nutritional strategies (59.3%), betablockers (44.4%), early coverage and grafting (40.7%) and anabolic steroids (37%). Less frequently reported interventions included exercise (11.1%) and infection control (11.1%).

#### Muscle wasting

Few clinicians reported the assessment of muscle wasting (15.3%). Of the methods used, indirect methods, such as body-weight monitoring (11.9%), eye judgement of muscle volume (5.1%) or muscle force measurement (3.4%), were most commonly mentioned. Only one clinician reported the use of bioimpedance analysis, nitrogen balance or muscle circumference measurements. Muscle force, in contrast, was assessed more commonly, with 59.4% of therapists reporting its use. Manual muscle testing using the common Medical Research Council scale of 0–5 points was used most frequently (46.9%) together with handheld dynamometry (31.3%) and indirect measures through functional tests (25%). In contrast, isokinetic dynamometry was carried out less frequently (3.1%).

Interventions to manage muscle wasting were reported by 70.4% of clinicians. These included exercise (66.7%) and nutritional adaptation (55.6%) as primary strategies, whereas the administration of anabolic steroids (14.8%) or betablockers (7.4%) were less frequently reported.

#### Insulin sensitivity

Measurement of insulin sensitivity in burn patients was not widespread, with only two medical doctors reporting its use. The insulin indices calculated were the homeostasis model assessment of insulin resistance (HOMA-IR) and the insulin sensitivity index (ISI). Insulin sensitivity was reported as a therapeutic target by 44.4% of respondents. A large variation in intervention strategies to manage the development of insulin resistance was noted. The main strategy of choice consisted of the infusion of insulin (48.1%), with glycaemic targets split between moderate or tight glycaemic control at 25.9% and 22.2%, respectively. Among the less-frequently-stated interventions were exercise, a hypoglycaemic diet and the avoidance of overfeeding (each 7.4%).

### Priorities, motivation and knowledge

#### Treatment priorities

**Figure 2.**
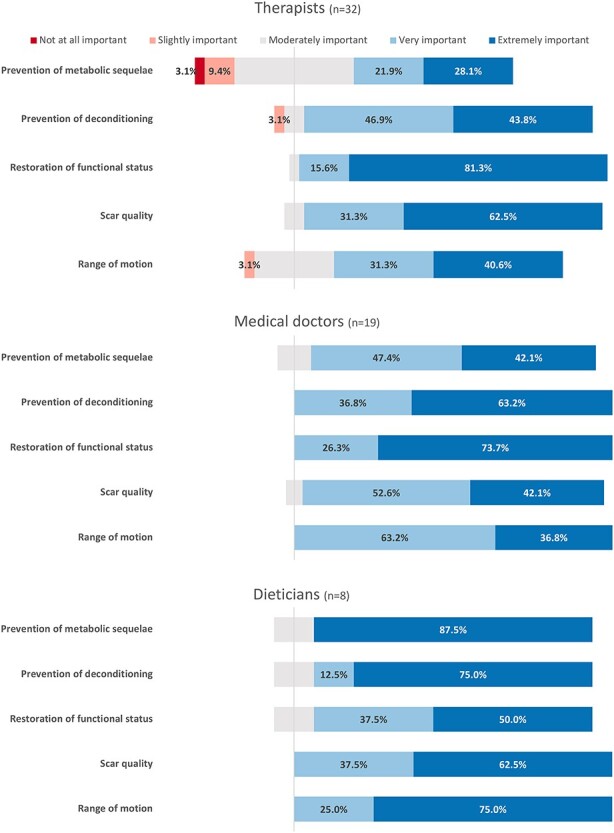
Importance ratings of treatment goals per profession

Responses showed interdisciplinary variations in the rating of importance (Figure 2). Most notable variations were seen in ratings of the restoration of functional status and the prevention of metabolic sequelae, with the former receiving the highest importance scores amongst medical doctors and therapists, yet the lowest importance scores by dieticians. Similarly, the latter group gave the highest importance to the prevention of metabolic sequelae, while medical doctors and therapists rated it as being of lowest importance (Figure 2). Dieticians were nearly 18 times more likely than therapists to rate the prevention of metabolic sequelae as extremely important (OR, 17.89; 95% CI, 1.92–166.78; *p* < 0.01) (Table S4).

#### Rationale for active exercise

A similar sequence of priorities was found when therapists were asked to list reasons (in descending priority) why they thought active exercise should be included in the acute phase of burns. The given reasons and their assigned priority varied considerably among therapists, with the restoration of functional status (78.1% of respondents) and preservation of joint range of motion (53.1%) being the most frequently mentioned. Psychological and motivational effects (34.4%), cardiovascular fitness (34.4%), muscle strength (31.1%) and the restoration of muscle mass (28.1%) were listed as reasons for active exercise less often. Besides the restoration of muscle mass, no mention was made of other potential metabolic effects of exercise, such as glycaemic control.

#### Knowledge of burn-induced metabolic effects

**Figure 3.**
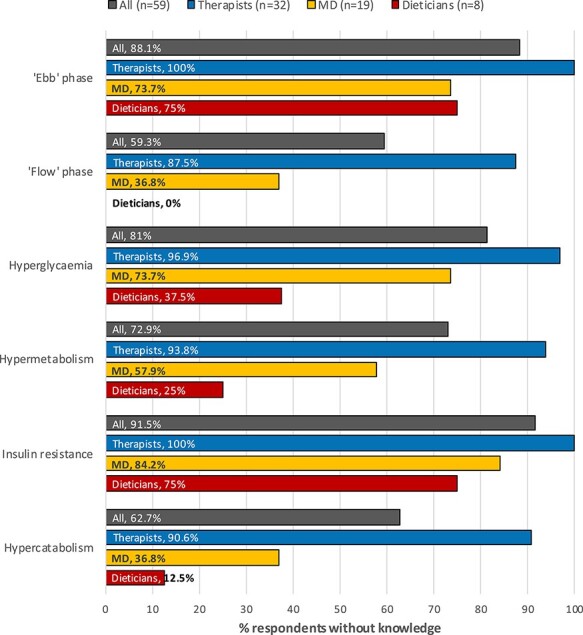
Percentage of respondents unable to correctly identify respective metabolic sequelae of burns. *MD* medical doctor

When asked to list the short- and long-term metabolic effects of major burns, few burn clinicians were able to correctly identify the ebb phase (11.9%) or any components of the flow phase (40.7%), including the potential development of hypermetabolism (27.1%), hyperglycaemia (18.6%), insulin resistance (8.5%) and hypercatabolism (37.3%). When divided into subgroups according to discipline, therapists demonstrated the least knowledge of metabolic sequelae, with none able to identify the ebb phase, and only 4 respondents (12.5%) correctly stating at least one component of the flow phase (Figure 3). Medical doctors were 12 times more likely than therapists to be able to identify at least one component of the flow phase (OR, 12.00; 95% CI, 2.95–48.78; *p* < 0.01) (Table S4). While more medical doctors and dieticians correctly identified post-burn metabolic sequelae, a significant number nonetheless were unable to do so, with the majority of respondents (12 medical doctors, 84.2%, 6 dieticians, 75%) not listing the potential development of insulin resistance as a metabolic sequela. Overall, being able to identify at least one component of the flow phase quadrupled the odds of assigning the highest importance rating to the prevention of metabolic sequelae (OR 4.63; 95%CI 1.50-14.25; p < 0.01) (Table S4).

## Discussion

This study surveyed burn clinicians across European burn centres to examine current inpatient management of adults with moderate to severe burns with respect to metabolic outcomes, exercise prescription, treatment priorities and knowledge of burn-induced metabolic sequelae. The survey revealed considerable non-uniformity in multiple aspects of current rehabilitation practice. Our main findings indicate that resistance and aerobic exercise in particular are not invariably administered, and that burn-induced metabolic sequelae appear to be neglected as both assessment and therapeutic targets in the inpatient management of adults with moderate to severe burns across Europe.

### Exercise prescription

Our data demonstrates that exercise is administered by all surveyed therapists at some stage of inpatient recovery. However, large variability was present between therapists in the timing of exercise initiation, the use of inclusion/exclusion criteria for exercise and exercise parameters. The majority of therapists stated that they did not categorically provide resistance or aerobic exercise to patients while intubated, and, for some, this remained in effect until patients were allowed to leave their hospital rooms. It is during the early phase of recovery that burn-induced metabolic derangements and prolonged inactivity are most prominent and combine to cause unwanted effects such as muscle wasting and glucose intolerance [2, 4, 5, 38]. Maximum exercise stimuli during this early phase, in particular through resistance and aerobic exercise, would seem most intuitive to lessen the negative sequelae of the metabolic imbalance [22, 42]. However, our data indicates that early exercise in European burn care does not categorically include resistive and aerobic components, both of which appear secondary to range-of-motion or functional exercise components.

Few other studies have surveyed inpatient rehabilitation in burns [34–36, 41, 43–46], of which two reports investigated the use of resistance and aerobic exercise in the acute phase of both adult and paediatric burns [35, 36]. Cambiaso-Daniels *et al.* reported that resistance and aerobic exercise was offered to all patients (mixed adult and paediatric burns) admitted to ICUs of six major American burn centres [35]. Our results differ from theirs in that not all our respondents stated using both resistance and aerobic exercises across all phases of inpatient stay. Instead, we were able to show a progression in resistance and aerobic exercise provision depending on the stage of recovery (Table 1). Such a progression is in agreement with the findings of another recent report by Flores *et al*., which provides an excellent overview of exercise use throughout the entire recovery continuum worldwide and reports the pooled results of both adult and paediatric burns [36]. Their reported results show an increased provision of resistance and aerobic exercise after ICU discharge, with the majority of respondents using resistive (79.3%) and aerobic (71%) exercise components in the later recovery stage after wound closing. Such an observed progression is likely a reflection of the limited capacity of these patients to engage in active exercise, which generally improves over time throughout their stay in the burn centre. Although patient participation is an important factor to consider [25] and active exercise might be relatively time-intensive, early exercise provision remains critical and should neither be delayed nor compromised on [25, 28].

Traditionally, inpatient burn rehabilitation has focused on the skin, return to function and joint mobility, with guidelines for both adult and paediatric burns primarily concerned with positioning, splinting and scar management [21, 47, 48]. Only recently have guidelines concerning adult and paediatric rehabilitation included advice regarding inpatient exercise [20, 22, 25, 49, 50], albeit largely without concrete recommendations as to specific exercise parameters, such as exercise components or starting criteria. The lack of reported use of resistance and aerobic exercise therefore likely mirrors the equivalent lack of international, national and/or institutional exercise guidelines for severely burned patients in the acute phase. A survey conducted among burn clinicians treating patients of all ages across the US, Canada, Australia and New Zealand indeed showed that not all have guidelines to follow for their inpatient treatment [44]. While our survey did not assess the use of guidelines, a similar situation across European burn centres might hamper consistent exercise provision.

The majority of respondents of our survey stated not using any inclusion/exclusion criteria for active exercise. In addition, the choice of criteria differed greatly between clinicians, which is in agreement with the findings of a previous report investigating both adult and paediatric rehabilitation practices [36]. These observations might explain why more active exercise is not currently carried out at an earlier time point throughout patient recovery. Using clearly defined criteria to determine when and in whom exercise can be safely carried out is paramount to encouraging early targeted exercise provision [49]. Recommendations for safety criteria for commencing exercise in critically ill adults have been published in the intensive care literature [51, 52]. Such recommendations are needed for the burn population and should include the formulation of clearly defined inclusion and exclusion criteria.

Another component of exercise prescription that still requires consensus recommendations is the methods to determine the intensity of aerobic and resistance exercise. Our data shows that methods varied considerably between clinicians, with the vast majority using subjective methods, such as patient tolerance, to determine the intensity. This observation is paralleled by two previous surveys of both adult and paediatric rehabilitation practices [35, 36] and highlights the discordance between research and clinical practice in the use of objective methods for the determination and progression of exercise intensity.

Evidence for the effects of early exercise approaches in adults, including resistive and aerobic components, has been firmly established in other critical illnesses, with favourable effects on a multitude of outcomes, such as muscle strength, duration of mechanical ventilation, ICU length of stay, hospital length of stay, and hospital mortality [28, 29, 53]. This is in stark contrast to the burn population, in which, to the best of our knowledge, only one trial has assessed the effect of early mobilization techniques in adult burn ICUs prospectively [54]; two retrospective trials have been reported [55, 56]. Positive outcomes reported include reductions in ICU length of stay, hospital length of stay improvements in joint range of motion and fewer complications and contractures [54–56]. Despite solid evidence from the intensive care literature and preliminary evidence in the burn population, our results indicate that the practice of early exercise is not consistently implemented in the current acute care of burn survivors in Europe.

### Metabolic management

Burn patients undergo unparalleled surges in metabolic rate, protein catabolism and levels of insulin and fasting glucose. These metabolic changes have been shown to persist long after the initial trauma and produce impactful sequelae, such as loss of lean mass and insulin sensitivity, placing the burn survivor at an increased risk of long-term morbidity [7, 9, 11, 57].

Strategies to modulate the metabolic response and its sequelae during the acute phase of burns are thus invaluable to full recovery and rehabilitation [2]. The results of this study show large heterogeneity among surveyed burn clinicians in the use and choice of interventions used to manage the development of hypermetabolism, muscle wasting and insulin resistance (Table 4). A considerable number of respondents reported no specific strategy at all. Moreover, while exercise therapy was the main strategy of choice to counteract muscle wasting, very few opted for exercise as a strategy to manage insulin resistance or the hypermetabolic response.

Exercise-based interventions have been shown to mitigate muscle wasting and insulin resistance, as evidenced in healthy adults and patients with diabetes, as well as the critically ill [29, 32, 33, 58–60]. In burns, the potential of exercise to induce positive effects on energy expenditure, muscle mass and insulin sensitivity has also been investigated, albeit largely in the post-discharge phase and predominantly in paediatric patients [16, 18, 20, 23, 24, 61]. However, the use of exercise for these purposes in adults as part of inpatient care continues to be a largely unexplored, yet promising, area of future research [62]. Our data shows that metabolic outcomes are not consistently used as therapeutic exercise targets and that more guidance is needed to reach consensus about the role exercise can play in the metabolic management of burn patients.

The monitoring of metabolic outcomes provides invaluable information as to the effects of interventions and patient recovery. However, the results of this survey show that the assessment of energy expenditure, muscle wasting, muscle strength and insulin sensitivity was not widespread among respondents, and large variability was observed in employed methods of assessment (Table 3). Nutritional guidelines for major burns recommend the use of indirect calorimetry to match caloric provision with caloric requirements in all age groups [63]. However, energy expenditure was not measured by the majority of responding clinicians, but rather predicted via equations, paralleling the findings of a previous European survey in regard to the nutritional management of adult burn patients [19].

Similarly, the vast majority of respondents reported not measuring insulin sensitivity or muscle wasting, indicating that clinicians either lacked available tools of assessment or perceived the assessment of metabolic outcomes as less important. Dual X-ray absorptiometry, computed tomography and magnetic resonance imaging, histological analysis of muscle specimens, stable isotope infusions and the urea-to-creatinine ratio are excellent methods most commonly used in research to determine the degree of muscle wasting in critically ill adults and children [64–68]. The invasive or cost- and time-intensive nature of many of these methods, however, likely hinders their implementation into clinical practice. Other novel and promising methods that have been developed for the use in the ICU setting are bioelectrical impedance and musculoskeletal ultrasound [69–71]. Both are practical, non-invasive, bedside tools that have shown to be valid and related to a variety of clinical outcomes, such as mortality and morbidity in critically ill adults [72–80]. Their usefulness in assessing muscle parameters in burn patients has yet to be evaluated.

Likewise, the assessment of insulin sensitivity is clinically relevant, as persisting insulin resistance poses a significant challenge to the long-term health of survivors [7, 38, 81]. In the absence of hyperinsulinaemic euglycaemic clamp or oral glucose tolerance testing, valuable estimates of insulin resistance can be derived via simple indices, such as the homeostatic model assessment [82].

Consensus has yet to be established as to whether insulin sensitivity, as well as muscle wasting and energy expenditure, should be routinely measured as part of standard care, or whether certain criteria should indicate its use.

### Priorities, motivation and knowledge

Clinical decision making involves weighing competing priorities according to perceived importance of treatment goals. Successful burn rehabilitation also includes tailoring therapy to the individual needs of the patient [22]. While this may, at times, require therapy provision to focus more on one treatment goal, it should not lead to a systematic neglect of another.

Our results indicate that the therapist’s primary efforts are directed at the preservation of range of motion and restoration of functional status, and not burn-induced metabolic sequelae, including hypermetabolism, hypercatabolism and insulin resistance. It is then not surprising that resistance and aerobic exercise were not invariably administered at the early stages by the respondents of this survey. The reason why burn therapists perceive burn-induced metabolic sequelae as less important remains unclear. It is striking, however, that this observation was paralleled by a substantial lack of understanding of metabolic sequelae among all clinicians. While there are no standards as to the “right” priorities and time allocation of treatment components, it seems concerning that a limited insight into metabolic sequelae after burns may have contributed to a lower assigned priority. Therapists in particular, as the primary provider of exercise [44], need to be able to engage in informed clinical decision making based on the perceived importance of treatment goals—a rather uninformed understanding of the metabolic sequelae and their impact on the burn survivor would certainly contribute to an inadequate management of them.

The ability to describe the physiological responses to increased activity, as well as the knowledge of indications and rationale for aerobic and resistance exercise are both described as core competencies in the burn rehabilitation therapist competency tool by the American Burn Rehabilitation Committee, concerning the treatment of all age groups [83]. In Europe, such knowledge core competencies are missing, and the results of this survey indicate that the adherence to such competencies would be challenging [25]. Qualitative research in the adult ICU setting has identified the expectations and knowledge (including rationale for rehabilitation, perceived benefits and experience) of clinicians as being a primary barrier to implementation of early exercise rehabilitation [84]. In a survey of Chinese burn centres for both adult and paediatric burns, Chen *et al*. likewise identified insufficient knowledge of burn clinicians as a primary factor impeding early rehabilitation practice in China [45]. Our findings indicate that such a knowledge barrier may indeed have played a role in the survey responses for two reasons. First, we found a significant positive association between the understanding of metabolic sequelae and their importance ratings. Secondly, exercise prescription remained unchanged in the face of post-burn metabolic sequelae due to a reported lack of understanding of metabolic sequelae and the effects of exercise on these parameters.

Therapists are classically trained in the musculoskeletal domain, whereas metabolic and internal disorders generally lie within the field of expertise of medical doctors and dieticians. Such a division, however, appears problematic as exercise therapy does play an important role in the management of burn-induced metabolic sequelae. Issues such as increased muscle catabolism and glucose intolerance present particularly promising therapeutic targets that ought not to be neglected during the acute phase of critical illness [15, 22, 33]. It needs to be emphasized that successful early exercise rehabilitation involves the entire multidisciplinary team [25, 84, 85]. The results of this survey show substantial interdisciplinary variations in both the understanding and importance of metabolic sequelae. To align treatment priorities across disciplines, a concomitant aligning of the understanding of metabolic sequelae becomes imperative.

This is the first study to survey burn clinicians with respect to inpatient rehabilitation exclusively for adult burns. Previous surveys have pooled responses for the rehabilitation of paediatric and adult patients, or had a different scope (post-grafting mobility, post-discharge rehabilitation [34–36, 41, 43–46]). According to our best knowledge, the present survey also forms the first of its kind to investigate burn rehabilitation with a focus on metabolic outcomes and the knowledge and priorities of clinicians. As such, it points to the necessity of education, or re-education, within the field of burn-induced metabolic sequelae for burn clinicians (therapists in particular) and draws out particular areas in need of attention for future practice guidelines.

### Limitations

Several limitations can be identified in our study. One major shortcoming of this survey is its limited reach to only European countries, as well as burn clinicians not reached due to inactive or absent email addresses. This may have introduced potential nonresponse bias. Despite sending a reminder email, the response rate primarily relied on the initial recipient forwarding the email to the respective burn clinicians. As it is unclear how many overall eligible burn clinicians were active in European burn centres at the time of data collection, we were unable to determine the external validity of our sample. The results of this survey may therefore not adequately represent the entire European context, instead only providing a snapshot of selected countries and responding participants. The overall response rate may nonetheless be underestimated as it is uncertain how many of the email contacts were active at the point of distribution.

A second major shortcoming is that multiple surveys from the same burn profession at the same burn centres were allowed. While this slightly inflated the burn-centre-to-survey ratio, we opted for this strategy as we anticipated differing responses within the same discipline. Our primary interest was to investigate variability of current practice between burn clinicians, as opposed to between-centre variability.

Third, while we show that metabolic sequelae were neither widely understood nor commonly considered as therapeutic exercise targets by the majority of surveyed burn clinicians, this study was not designed to test a cause–effect relationship between the clinician’s understanding and specific choices of treatment. Nonetheless, qualitative research into barriers of early exercise in the wider critically ill population points to the clinician’s knowledge as a major barrier to implementation [84]. Whether this observation also holds true in the burn population remains to be confirmed.

Last, it is possible that differences in the responses given by burn clinicians might not fully represent true variability between respondents, but rather be attributed to differences in admission rate or burn centre size. We were unable to control for the number of admissions or size of the burn facilities, as the developers of the survey purposefully chose not to ask for this information. This decision was made in the hope of a higher response rate by minimizing the risk that respondents would abandon survey completion due to being unable to answer these early questions without consulting other administrative staff.

## Conclusions

Burn-induced metabolic sequelae are important rehabilitation targets in the successful management of burns. Although early exercise rehabilitation has the potential to significantly alter the trajectory of metabolic sequelae of burn survivors, the results of this survey demonstrate that considerable nonuniformity exists around its provision across European burn care. This survey reveals a potential neglect of burn-induced metabolic sequelae as therapeutic and assessment targets, which might be grounded in a limited understanding of metabolic pathophysiology. Overall, our results reflect the paucity of scientific research into the effects of early exercise on metabolic outcomes in the adult burn population, and point to the need for well-designed trials to pave the way for more conformity in the acute care of burn survivors. Future direction and guidance should focus on: (1) further defining the role of metabolic outcomes as rehabilitation targets; (2) establishing core competencies for rehabilitation staff in Europe, including the rationale for resistance and aerobic exercise; and (3) further investigating barriers and enablers to implementing successful early rehabilitation of burn survivors.

## Supplementary Material

S1_tkaa039

S2-S4_tkaa039

## Data Availability

All data generated or analysed during the current study is included in this manuscript.
