# Supplementary material for: Status of adult inpatient burn rehabilitation in Europe: are we neglecting metabolic outcomes?
Source: Burns Trauma. 2021 Mar 1;9:tkaa039. doi: 10.1093/burnst/tkaa039 (PMC7935379; doi:10.1093/burnst/tkaa039)
Supplement: S1_tkaa039 [file s1_tkaa039.docx]

S1. Copy of Survey

***** denotes forced-response question

**QUESTIONS FOR ALL PROFESSIONS:**

Q1. Affiliated burn centre:* *Please enter text here*

Q2. What is your profession?*

- Physiotherapist
- Occupational therapist
- Dietician
- MD
- Nurse

Q3. How many years of professional experience do you have in burns?*

Years: *Please enter text here*

Q4. Do you work in inpatient burn care (i.e. stationary patients)?*

- Yes
- No

Q5. What age group of burn patients do you treat?*

- adult (≥18 years)
- children (<18 years)
- all ages

Q6. Currently, very little is known about the (patho-)physiological short- and long-term metabolic effects of severe burns (≥20%TBSA) in adult patients. Do you know anything about these effects?*

- No idea
- Yes (you will be asked to specify in the next question)

Q7. Please list as many (patho-)physiological short- and long-term metabolic effects of severe burns (≥20%TBSA) in adult patients as you can think of below:

*Please answer this question without consulting other knowledge resources. The aim of this question is to get a realistic picture of the readily available knowledge (that which you can recall without any additional resources) which informs daily clinical decision-making.*

Short-term (<24-72 hours postburn):

| (1) | *Please enter text here* |
| --- | --- |
| (2) | *Please enter text here* |
| (3) | *Please enter text here* |
| (4) | *Please enter text here* |
| (5) | *Please enter text here* |
| (6) | *Please enter text here* |
| (7) | *Please enter text here* |
| (8) | *Please enter text here* |
| (9) | *Please enter text here* |
| (10) | *Please enter text here* |

Long-term (>24-72 hours postburn):

| (1) | *Please enter text here* |
| --- | --- |
| (2) | *Please enter text here* |
| (3) | *Please enter text here* |
| (4) | *Please enter text here* |
| (5) | *Please enter text here* |
| (6) | *Please enter text here* |
| (7) | *Please enter text here* |
| (8) | *Please enter text here* |
| (9) | *Please enter text here* |
| (10) | *Please enter text here* |

**All following questions concern the acute phase (i.e. in-hospital stay) of severely burned adults (≥20%TBSA)**

Q8. How important are the following treatment goals for severely burned adult patients (≥20%TBSA) over the entire hospital stay in your opinion?*

*(on a scale from "not at all important" to "extremely important")*

|  | Not at all important | Slightly important | Moderately important | Very important | Extremely important |
| --- | --- | --- | --- | --- | --- |
| Range of motion (joint mobility, skin mobility) |  |  |  |  |  |
| Scar quality (aesthetics, pruritus, pain, prevention of hypertrophic scarring, etc.) |  |  |  |  |  |
| Restoration of functional status (ADL's, ambulation ability, etc.) |  |  |  |  |  |
| Prevention of deconditioning (muscle weakness, cardiovascular deconditioning, etc.) |  |  |  |  |  |
| Prevention of metabolic sequelae (insulin resistance, hypermetabolism, hyperglycaemia, fat and muscle catabolism, etc.) |  |  |  |  |  |

Q9. Do you measure loss of muscle mass (i.e. muscle wasting) in adult burn patients (≥20%TBSA) in the acute phase (in-hospital stay)?*

- Yes
- No

Q10. How do you measure loss of muscle mass (i.e. muscle wasting) in adult burn patients (≥20%TBSA) in the acute phase (in-hospital stay)?* *(Multiple answers possible)*

- DXA scan
- MRI scan
- CT scan
- Musculoskeletal ultrasound
- Bioimpedance
- Nitrogen balance
- Indirectly through muscle strength
- Muscle circumference measures
- Eye judgement of muscle volume
- Other (please specify): *Please enter text here*

Q11. How regularly do you measure loss of muscle mass (i.e. muscle wasting) in adult burn patients (≥20%TBSA) in the acute phase (in-hospital stay)?*

Daily

- Weekly
- Biweekly
- Only when indicated (please specify): *Please enter text here*

**ADDITIONAL QUESTIONS FOR THERAPISTS:**

Q12. Do you include active exercise as part of the adult burn rehabilitation (≥20%TBSA) in the acute phase (in-hospital stay)?*

*(Active exercise = independent or assisted movements / contractions of the patients' muscles)*

- Yes
- No
- Sometimes

Q13. How do you determine whether active exercise training is indicated for adult burns (≥20%TBSA) in the acute phase (in-hospital stay)?* *(Active exercise = independent or assisted movements / contractions of the patients' muscles)*

*(Multiple answers possible)*

- I use predefined in-/exclusion criteria
- I do NOT use in-/exclusion criteria
- By doctors' prescription only

Q14. Which in-/exclusion criteria do you use to decide when active exercise training in the acute phase (in-hospital stay) is indicated for adult burns (≥20%TBSA)?* *(Active exercise = independent or assisted movements / contractions of the patients' muscles)*

- %TBSA
- Neurological stability
- Cardiorespiratory stability
- Breathing status
- Acute surgery
- Temperature
- Level of cooperation
- Level of alertness
- Muscle strength
- Other (please specify): *Please enter text here*

Q15. Do you use aerobic training as part of your exercise training in the acute phase (in-hospital stay) for adult burns (≥20%TBSA)?*

- Yes
- No
- Sometimes

Q16. How do you determine the intensity of the aforementioned aerobic training?*

*​(Multiple answers possible)*

- Heart Rate
- VO2 max
- General exercise guidelines (please specify): *Please enter text here*
- Patient Tolerance
- Other (please specify): *Please enter text here*

Q17. Do you use strength training as part of your exercise training in the acute phase (in-hospital stay) for adult burns (≥20%TBSA)?*

- Yes
- No
- Sometimes

Q18. How do you determine the intensity of the aforementioned strength training?*

*(Multiple answers possible)*

- Manual muscle testing
- Dynamometry
- RM's (repetition maximum)
- Patient tolerance
- Other (please specify): *Please enter text here*

Q19. Which muscle groups do you generally train during the aforementioned strength training in the acute phase (in-hospital stay) for adult burns (≥20%TBSA)?*

*(Multiple answers possible)*

- Whole body
- Lower limbs
- Upper limbs
- Core
- Other (please specify): *Please enter text here*

Q20. What therapy components do you generally train when adult burn patients (≥20%TBSA) are intubated, and how much % of total treatment duration does each represent approximately?*

*Write 0% when you do not train a particular component, total must amount to 100%*

*If you do not treat the intubated at all, please write: "not applicable" in the "other" box and assign 100% to it.*

| Range of Motion | 0% |
| --- | --- |
| Aerobic | 0% |
| Strength | 0% |
| Proprioception | 0% |
| Function | 0% |
| Respiratory | 0% |
| Other (please specify): *Please enter text here* | 0% |
| **Total** | 100% |

Q21. What therapy components do you generally train when adult burn patients (≥20%TBSA) are NOT able to leave their room, and how much % of total treatment duration does each represent approximately?*

*Write 0% when you do not train a particular component, total must amount to 100%*

*If you do not treat these patients at all, please write: "not applicable" in the "other" box and assign 100% to it.*

| Range of Motion | 0% |
| --- | --- |
| Aerobic | 0% |
| Strength | 0% |
| Proprioception | 0% |
| Function | 0% |
| Respiratory | 0% |
| Other (please specify): *Please enter text here* | 0% |
| **Total** | 100% |

Q22. What therapy components do you generally train when adult burn patients (≥20%TBSA) are able to leave their room, and how much % of total treatment duration does each represent approximately?*

*Write 0% when you do not train a particular component, total must amount to 100%*

*If you do not treat these patients at all, please write: "not applicable" in the "other" box and assign 100% to it.*

| Range of Motion | 0% |
| --- | --- |
| Aerobic | 0% |
| Strength | 0% |
| Proprioception | 0% |
| Function | 0% |
| Respiratory | 0% |
| Other (please specify): *Please enter text here* | 0% |
| **Total** | 100% |

Q23. Do you measure muscle strength in adult burn patients (≥20%TBSA) in the acute phase (in-hospital stay)?*

- Yes
- No

Q24. How do you measure muscle strength in adult burn patients (≥20%TBSA) in the acute phase (in-hospital stay)*?**

*(Multiple answers possible)*

- Manual Muscle Testing (Medical Research Council)
- Hand-held dynamometry
- Isokinetic dynamometry (e.g. Biodex)
- Indirectly through functional tests
- Other (please specify): *Please enter text here*

Q25. How regularly do you measure muscle strength in adult burn patients (≥20%TBSA) in the acute phase (in-hospital stay)?*

- Daily
- Weekly
- Biweekly
- Only when indicated (please specify): *Please enter text here*

Q26. How long does the overall exercise programme for adult burn patients (≥20%TBSA) generally last?*

- For ___ weeks (please specify)
- until discharge from intensive care
- until discharge from burn unit
- until discharge from hospital
- until after discharge from hospital
- until goals are achieved
- Other (please specify): *Please enter text here*

Q27. Do you advise adult burn patients (≥20%TBSA) to follow an exercise programme after hospital discharge?*

- Yes
- No
- Depends on the patient

Q28. Why, according to you, should exercise training be included in the acute phase of severe burns (≥20%TBSA)?

*List as many reasons as you can think of according to priority. (1 = highest priority)*

*If you cannot think of any, leave the fields empty.*

| (1) | *Please enter text here* |
| --- | --- |
| (2) | *Please enter text here* |
| (3) | *Please enter text here* |
| (4) | *Please enter text here* |
| (5) | *Please enter text here* |
| (6) | *Please enter text here* |
| (7) | *Please enter text here* |
| (8) | *Please enter text here* |
| (9) | *Please enter text here* |
| (10) | *Please enter text here* |

Q29. Does the development of insulin resistance, hyperglycaemia, hypermetabolism (i.e. >10% increased metabolic rate above predicted) in severely burned adults (>20%TBSA) change your exercise prescription in the acute phase (in-hospital stay)?*

*(Multiple answers possible)*

- Yes
- No, this is the responsibility of doctors, dietists, intensivist
- No, I don't know enough about metabolic sequelae after burns
- No, I wouldn't know how to change the exercise prescription accordingly
- Other (please specify): *Please enter text here*

**ADDITIONAL QUESTIONS FOR MEDICAL DOCTORS / DIETICIANS:**

Q30. How do you determine energy expenditure / caloric requirements for adult burn inpatients (>20%TBSA)?*

*(Multiple answers possible)*

- Prediction formulas (please specify): *Please enter text here*
- Indirect calorimetry
- Other (please specify): *Please enter text here*

Q31. How regularly do you use prediction formulas to determine energy expenditure / caloric requirements in adult burn inpatients (>20%TBSA)?*

- Daily
- Weekly
- Biweekly
- Only when indicated (please specify): *Please enter text here*

Q32. How regularly do you use indirect calorimetry to determine energy expenditure / caloric requirements in adult burn inpatients (>20%TBSA)?*

- Daily
- Weekly
- Biweekly
- Only when indicated (please specify): *Please enter text here*

Q33. How regularly do you use “value entered in text field Q31” to determine energy expenditure / caloric requirements in adult burn inpatients (>20%TBSA)?*

- Daily
- Weekly
- Biweekly
- Only when indicated (please specify): *Please enter text here*

Q34. Describe the methods you use for indirect calorimetry in adult burn inpatients (>20%TBSA).*

*(e.g. fasted state, face mask, ventilated hood method, through mechanical ventilation, duration of measurement, time point of the day, etc)*

*Please enter text here*

­­­Q35. Which intervention strategies do you use at your burn centre to manage the development of a hypermetabolic state (i.e. >10% of predicted resting energy expenditure) in adult burn patients (>20%TBSA) in the acute phase of burns (in-hospital stay)?

*(If you do not use any, leave the fields empty)*

| (1) | *Please enter text here* |
| --- | --- |
| (2) | *Please enter text here* |
| (3) | *Please enter text here* |
| (4) | *Please enter text here* |
| (5) | *Please enter text here* |
| (6) | *Please enter text here* |
| (7) | *Please enter text here* |
| (8) | *Please enter text here* |
| (9) | *Please enter text here* |
| (10) | *Please enter text here* |

Q36. Do you measure insulin sensitivity in adult burn patients (≥20%TBSA) in the acute phase (in-hospital stay)?*

- Yes
- No

Q37. How do you determine insulin sensitivity in adult burn patients (≥20%TBSA) in the acute phase (in-hospital stay)?*

*(Multiple answers possible)*

- Euglycemic Clamp
- OGTT (Oral Glucose Tolerance Test)
- HOMA (homeostatic model assessment)
- HOMA2 (improved model of HOMA)
- QUICKI (quantitative insulin-sensitivity check index)
- ISI (insulin sensitivity index)
- IGI (insulinogenic index)
- Other (please specify): *Please enter text here*

Q38. How regularly do you measure insulin sensitivity in adult burn patients (≥20%TBSA) in the acute phase (in-hospital stay)?*

- Daily
- Weekly
- Biweekly
- Only when indicated (please specify): *Please enter text here*

Q39. Which intervention strategies do you use at your burn centre to manage the development of insulin resistance in adult burn patients (>20%TBSA) in the acute phase of burns (in-hospital stay)?

*(If you do not use any, leave the fields empty)*

| (1) | *Please enter text here* |
| --- | --- |
| (2) | *Please enter text here* |
| (3) | *Please enter text here* |
| (4) | *Please enter text here* |
| (5) | *Please enter text here* |
| (6) | *Please enter text here* |
| (7) | *Please enter text here* |
| (8) | *Please enter text here* |
| (9) | *Please enter text here* |
| (10) | *Please enter text here* |

Q40. Which intervention strategies do you use at your burn centre to manage the loss of muscle mass (i.e. muscle wasting) in adult burn patients (>20%TBSA) in the acute phase of burns (in-hospital stay)?

*(If you do not use any, leave the fields empty)*

| (1) | *Please enter text here* |
| --- | --- |
| (2) | *Please enter text here* |
| (3) | *Please enter text here* |
| (4) | *Please enter text here* |
| (5) | *Please enter text here* |
| (6) | *Please enter text here* |
| (7) | *Please enter text here* |
| (8) | *Please enter text here* |
| (9) | *Please enter text here* |
| (10) | *Please enter text here* |
