# Supplementary material for: Status of adult inpatient burn rehabilitation in Europe: are we neglecting metabolic outcomes?
Source: Burns Trauma. 2021 Mar 1;9:tkaa039. doi: 10.1093/burnst/tkaa039 (PMC7935379; doi:10.1093/burnst/tkaa039)
Supplement: S2-S4_tkaa039 [file s2-s4_tkaa039.docx]

Table S2 ***Outcome measures*.** Data presented per profession.

| **Methods** | **% (frequency)** | | | | | | | | **Applied frequency % (frequency)** | | | | | | | | |
| --- | --- | --- | --- | --- | --- | --- | --- | --- | --- | --- | --- | --- | --- | --- | --- | --- | --- |
| **ENERGY EXPENDITURE** – *Responders:* Medical doctors & dieticians (n=27) | | | | | | | | | | | | | | | | | |
|  | MD | | Dietician | | Therapists | | TOTAL | | |  | | MD | | Dietician | | Therapists | TOTAL |
| Indirect calorimetry (IC)  - Via mechanical ventilator  - Spontaneous breathing  Criteria indicating use of IC:  - Mechanical ventilation  - %TBSA  - Unexplained weight loss  - Other metabolic issue | 25.9% (7)  25.9 % (7)  3.7% (1)  25.9% (7)  14.8% (4)  7.4% (2)  0% (0) | | 14.8% (4)  14.8% (4)  0% (0)  14.8% (4)  0% (0)  3.7% (1)  3.7% (1) | | /  /  /  /  /  /  / | | 40.7% (11)  40.7% (11)  3.7% (1)  40.7% (11)  14.8% (4)  11.1% (3)  3.7% (1) | | | Daily  Weekly  Biweekly  Only when indicated | | 3.7% (1)  14.8% (4)  0% (0)  7.4% (2) | | 0% (0)  11.1% (3)  0% (0)  3.7% (1) | | /  /  /  / | 3.7% (1)  25.9% (7)  0% (0)  11.1% (3) |
| Prediction formulas  - Toronto  - Fixed kcal/kg  - Harris Benedict  - Curreri  - Others^a^ | 63% (17)  22.2% (6)  7.4% (2)  3.7% (1)  7.4% (2)  7.4% (2) | | 25.9 % (7)  14.8% (4)  11.1% (3)  11.1% (3)  7.4% (2)  7.4% (2) | | /  /  /  /  /  / | | 88.9% (24)  37% (10)  18.5% (5)  14.8% (4)  14.8% (4)  14.8% (4) | | | Daily  Weekly Biweekly  Only when indicated | | 14.8% (4)  29.6% (8)  7.4% (2)  11.1% (3) | | 11.1% (3)  3.7% (1)  0% (0)  11.1% (3) | | /  /  /  / | 25.9% (7)  33.3% (9)  7.4% (2)  22.2% (6) |
| **MUSCLE WASTING** – *Respondents:* Medical doctors & dieticians & therapists (n=59) | | | | | | | | | | | | | | | | | |
|  | | MD | | Dietician | | Therapists | | TOTAL | | |  | | MD | | Dietician | Therapists | TOTAL |
| Not measured  Body weight monitoring  Eye judgement of muscle volume  Muscle force assessment  Muscle circumference  Nitrogen balance  Bio impedance analysis | | 22% (13)  14.8% (4)  1.7% (1)  1.7% (1)  0% (0)  1.7% (1)  1.7% (1) | | 10.2% (6)  3.4% (2)  1.7% (1)  1.7% (1)  1.7% (1)  0% (0)  0% (0) | | 52.5% (31)  1.7% (1)  1.7% (1)  0% (0)  0% (0)  0% (0)  0% (0) | | 84.7% (50)  11.9% (7)  5.1% (3)  3.4% (2)  1.7% (1)  1.7% (1)  1.7% (1) | | | Daily  Weekly  Biweekly  Only when indicated | | 0% (0)  10.2% (6)  0% (0)  0% (0) | | 0% (0)  1.7% (1)  0% (0)  1.7% (1) | 0% (0)  0% (0)  0% (0)  1.7% (1) | 0% (0)  11.9% (7)  0% (0)  3.4% (2) |
| **INSULIN SENSITIVITY** – *Respondents:* Medical doctors & dieticians (n=27) | | | | | | | | | | | | | | | | | |
|  | | MD | | Dietician | | Therapists | | TOTAL | | |  | | MD | | Dietician | Therapists | TOTAL |
| Not measured  HOMA-IR  ISI | | 63% (17)  3.7% (1)  3.7% (1) | | 29.6% (8)  0% (0)  0% (0) | | /  /  / | | 92.6% (25)  3.7% (1)  3.7% (1) | | | Daily  Weekly  Biweekly  Only when indicated | | 0% (0)  3.7% (1)  0% (0)  3.7% (1) | | 0% (0)  0% (0)  0% (0)  0% (0) | /  /  /  / | 0% (0)  3.7% (1)  0% (0)  3.7% (1) |
| **MUSCLE FORCE** – *Respondents:* Therapists (n=32) | | | | | | | | | | | | | | | | | |
|  | | MD | | Dietician | | Therapists | | TOTAL | | |  | | MD | | Dietician | Therapists | TOTAL |
| Not measured  Manual muscle testing  Handheld dynamometry  Indirectly through functional tests  Isokinetic Dynamometry | | /  /  /  /  / | | /  /  /  /  / | | 40.6% (13)  46.9% (15)  31.3% (10)  25% (8)  3.1% (1) | | 40.6% (13)  46.9% (15)  31.3% (10)  25% (8)  3.1% (1) | | | Daily  Weekly Biweekly  Only when indicated | | /  /  /  / | | /  /  /  / | 3.1% (1)  28.1% (9)  9.4% (3)  18.8% (6) | 3.1% (1)  28.1% (9)  9.4% (3)  18.8% (6) |

MD, medical doctors; HOMA-IR, homeostasis model assessment of insulin resistance; ISI, Insulin Sensitivity Index

^a^including Henry’s, Milner, Garland, Xi

Table S3. ***Metabolic interventions.*** Data presented per profession.

| **Therapeutic target** | **Intervention** | **% (frequency)** | | | |
| --- | --- | --- | --- | --- | --- |
|  |  | MD | Dietician | TOTAL |  |
| **Hypermetabolism**^a^  *Respondents:*  Medical doctors & dieticians (n=27) | No strategy  Modify nutrition^b^  Betablockers  Early coverage / grafting  Anabolic steroids  Glycaemic control  Early excision  Adapt ambient temperature  Exercise  Infection control  Others^c^ | 25.9% (7)  37% (10)  40.7% (11)  37% (10)  33.3% (9)  25.9% (7)  22.2% (6)  18.5% (5)  11.1% (3)  11.1% (3)  18.5% (5) | 14.8% (4)  22.2% (6)  3.7% (1)  3.7% (1)  3.7% (1)  3.7% (1)  3.7% (1)  3.7% (1)  0% (0)  0% (0)  0% (0) | 40.7% (11)  59.3% (16)  44.4% (12)  40.7% (11)  37% (10)  29.6% (8)  25.9% (7)  22.2% (6)  11.1% (3)  11.1% (3)  18.5% (5) |  |
| **Muscle Wasting**  *Respondents:*  Medical doctors & dieticians (n=27) | No strategy  Exercise  Modify nutrition  Anabolic steroids  Betablockers  Limit duration / depth of sedation  Others^d^ | 22.2% (6)  44.4% (12)  33.3% (9)  11.1% (3)  7.4% (2)  7.4% (2)  11.1% (3) | 7.4% (2)  22.2% (6)  22.2% (6)  3.7% (1)  0% (0)  0% (0)  0% (0) | 29.6% (8)  66.7% (18)  55.6% (15)  14.8% (4)  7.4% (2)  7.4% (2)  11.1% (3) |  |
| **Insulin Sensitivity**  *Respondents:*  Medical doctors & dieticians (n=27) | No strategy  Insulin infusion  Moderate glycaemic control  Tight glycaemic control  Hypoglycaemic diet  Avoid overfeeding  Anabolic steroids  Early excision  Exercise  Others^e^ | 33.3% (9)  25.9% (7)  11.1% (3)  14.8% (4)  0% (0)  3.7% (1)  3.7% (1)  0% (0)  0% (0)  18.5% (5) | 22.2% (6)  22.2% (6)  14.8% (4)  7.4% (2)  7.4% (2)  3.7% (1)  3.7% (1)  7.4% (2)  7.4% (2)  0% (0) | 55.6% (15)  48.1% (13)  25.9% (7)  22.2% (6)  7.4% (2)  7.4% (2)  7.4% (2)  7.4% (2)  7.4% (2)  18.5% (5) |  |

MD, medical doctors

^a^Defined as >10% predicted resting energy expenditure, ^b^including increasing and decreasing caloric provision, supplementing nutrition content (protein, trace elements, vitamins) early enteral feeding, ^c^including fenofibrates, growth hormones, early resuscitation, limiting sedation, anxiety reduction, ^d^including fenofibrates, avoiding neuromuscular blockers, early excision, early coverage, ^e^including Gliclazide, Metformin, betablockers, fenofibrates, early coverage

Table S4. ***Odds ratios.*** Data presented as odds ratio (95%CI).

|  | **MD vs. therapists** | **Dieticians vs.**  **therapists** | **Dieticians vs. MD** | **Knowledge of ‘flow’ phase**^a^ |
| --- | --- | --- | --- | --- |
| **Knowledge of the ‘flow’ phase**^a^ | 12.00 (2.95-48.78)  p<0.01 | / ^b^ | / ^b^ | / |
| **Importance ratings of prevention of metabolic sequelae**^c^ | 1.85 (0.56-6.13)  p=0.37 | 17.89 (1.92-166.78)  p<0.01 | 9.63 (0.98-94.54)  p=0.43 | 4.63 (1.50-14.25)  p<0.01 |
| **Importance ratings of prevention of deconditioning sequelae**^c^ | 2.20 (0.69-7.07)  p=0.25 | 3.86 (0.67-22.11)  p=0.24 | 1.75 (0.28-11.15)  p=0.68 | 2.25 (0.76-6.65)  p=0.18 |
| **Importance ratings of range of motion sequelae**^c^ | 0.85 (0.27-2.27)  p=1.00 | 4.39 (0.76-25.20)  p=0.12 | 5.14 (0.81-32.77)  p=0.10 | 1.91 (0.66-5.51)  p=0.29 |
| **Importance ratings of scar quality**^c^ | 0.44 (0.14-1.39)  p=0.24 | 1.00 (0.20-4.96)  p=1.00 | 2.29 (0.42-12.50)  p=0.42 | 0.70 (0.24-2.00)  p=0.60 |
| **Importance ratings of restoration of functional status**^c^ | 0.65 (0.17-2.50)  p=0.73 | 0.23 (0.05-1.20)  p=0.09 | 0.36 (0.06-2.00)  p=0.38 | 0.63 (0.19-2.11)  p=0.54 |

MD, medical doctors

^a^comparing present vs. absent ability to identify at least one component of the ‘flow’ phase in all respondents; ^b^no odds ratio could be computed as no dietician with absent knowledge was observed; ^c^comparing extremely important vs. all other importance ratings of respective therapy goals.
